# The *BH3 mimetic* HA14-1 enhances 5-fluorouracil-induced autophagy and type II cell death in oesophageal cancer cells

**DOI:** 10.1038/bjc.2011.604

**Published:** 2012-01-12

**Authors:** M J Nyhan, T R O'Donovan, B Elzinga, L C Crowley, G C O'Sullivan, S L McKenna

**Affiliations:** 1 Leslie C Quick Laboratory, Cork Cancer Research Centre, BioSciences Institute and Mercy University Hospital, University College Cork, Cork, Ireland

**Keywords:** oesophageal cancer, cell death, BH3 mimetic, HA14-1, autophagy, apoptosis

## Abstract

**Background:**

Resistance to chemotherapeutic agents has been associated with a failure of cancer cells to induce apoptosis. Strategies to restore apoptosis have led to the development of BH3 mimetics, which inhibit anti-apoptotic Bcl-2 family members. We examined the sensitivity of three oesophageal cancer cell lines to 5-fluorouracil (5-FU) alone and in combination with the BH3 mimetic HA14-1.

**Methods:**

Clonogenic assays, morphology, markers of autophagy and apoptosis were used to assess the involved death mechanisms.

**Results:**

In response to 5-FU treatment, OE21 cells induce apoptosis, KYSE450 and KYSE70 cells are more resistant and induce autophagy accompanied by type II cell death. Autophagy induction results in ineffective treatment as substantial numbers of cells survive and re-populate. HA14-1 did not improve 5-FU treatment or reduce colony re-growth in the apoptosis deficient KYSE70 cells. However, the sensitivity of OE21 (apoptotic) and KYSE450 cells (apoptosis deficient/type II cell death) was significantly improved. In OE21 cells, treatment with 5-FU and HA14-1 resulted in augmentation of apoptosis. In KYSE450 cells, the reduction in recovering colonies following combination treatment was due to the enhancement of type II cell death.

**Conclusion:**

The efficacy of HA14-1 is cell line dependent and is not reliant on apoptosis induction.

The prognosis of oesophageal cancer remains poor because of the absence of molecularly targeted therapies and resistance to conventional DNA-damaging chemotherapeutics. This resistance to therapy has been associated with a failure to induce apoptosis in response to DNA damage (O’Donovan *et al*, 2011).

Disruption of apoptotic signalling can be achieved by loss of tumour-suppressor activity and upregulation of survival signalling pathways (Adams and Cory, 2007). Ultimately, many signalling pathways that determine cell fate converge at the Bcl-2 family. In cancer, the balance between Bcl-2-negative regulators (Bcl-2/Bcl-x_L_/Mcl-1/A1/BCL-w) and positive regulators (Bax/Bak/BH3-only proteins) are disturbed such that initiation of apoptosis is difficult to achieve. Strategies to restore apoptosis have led to the development of BH3 mimetics (a novel class of therapeutics – now in clinical trials) designed to inhibit the interaction between Bcl-2 family members at BH3 domains (Zhang *et al*, 2007; Lessene *et al*, 2008; Kang and Reynolds, 2009).

Although this strategy is designed to engage the apoptosis pathway in otherwise resistant cells, both Bcl-x_L_ and Bcl-2 have also been implicated in the autophagy process. Both proteins can bind Beclin 1 (an autophagy regulator with a BH3 domain). Disruption of this interaction with ABT-737 (BH3 mimetic) induces autophagy (Maiuri *et al*, 2007). The BH3-only proteins Bid, Bad and BNIP3 have also been reported to induce autophagy in cancer cells (Lamparska-Przybysz *et al*, 2005; Hamacher-Brady *et al*, 2007; Maiuri *et al*, 2007). ApoL1, an autophagy regulator has a BH3 domain and its overexpression induces autophagic cell death (AuCD) (Wan *et al*, 2008).

Autophagy is a survival mechanism that enables cells to tolerate adverse conditions such as starvation or withdrawal of survival signalling. However, it also has the potential to drive type II cell death/AuCD (Berry and Baehrecke, 2007; Wan *et al*, 2008). It is possible therefore, that the cytotoxic effects of BH3 mimetics may not be limited to apoptosis induction.

In this study, we evaluated the effects of HA14-1 (BH3 mimetic) on three human squamous oesophageal cancer cell lines. Two of these cell lines fail to undergo apoptosis in response to 5-fluorouracil (5-FU) but instead induce an autophagic response, which is accompanied by type II cell death. Despite the presence of type II cell death morphologies, these populations will retain a substantial number of surviving autophagic cells that will re-populate, thus rendering treatment ineffective (O’Donovan *et al*, 2011). In this study, we evaluated the potential of HA14-1 to reduce this survival and assessed the involved death mechanism.

## Materials and methods

### Cell culture

Human oesophageal cancer cell lines KYSE450 and KYSE70 were from DSMZ (Deutsche Sammlung von Mikroorganismen und Zellkulturen GmbH, Braunschweig, Germany). OE21, OE19 and OE33 cells were from the European Collection of Cell Cultures, Salisbury, UK. KYSE450 cells were maintained in 50 : 50 RPMI 1640 : F-12 Hams medium, KYSE70, OE21, OE19 and OE33 cells were maintained in RPMI 1640 medium, all supplemented with 1% penicillin/streptomycin and 10% (v/v) fetal calf serum (Sigma-Aldrich, Arklow Co., Wicklow, Ireland) and grown at 37 °C, 5% CO_2_.

### Morphological examination of cell death

Morphological features of cells treated with 5-FU and the BH3 mimetic HA14-1 (Sigma-Aldrich) were examined by light microscopy. Cytospun cells were stained with Rapi-Diff (Braidwood Laboratories, Ringmer, East Sussex, UK). Apoptosis is characterised by cell shrinkage, chromatin condensation, DNA fragmentation into ‘apoptotic bodies’ within an intact plasma membrane. Type II cell death was identified by clear elevation of cytoplasmic vesicles, loss of cytoplasmic material, pyknosis of the nuclear material and an intact nuclear membrane (Clarke, 1990).

### Western blot analysis

Protein extracts were lysed in modified RIPA buffer (50 mM HEPES, 150 mM NaCl, 2 mM EDTA, 0.1-1% Nonidet P-40, protease inhibitor mix cocktail). Primary antibodies were: anti-Mcl-1, anti-Bcl-2, anti-Bax (Santa Cruz Biotechnology, Heidelberg, Germany); anti-Bcl-x_L_ (BD Transduction Laboratories, Oxford, UK) and anti-*β*-actin (Millipore, Carrigtwohill Co., Cork, Ireland). Proteins were visualised using relevant IR-DYE secondary antibodies (Rockland, Gilbertsville, PA, USA) on the Odyssey IR imaging system (Li-Cor, Cambridge, UK).

### Genechip array analysis

Affymetrix (High Wycombe, UK) gene array analysis of OE19, OE33, OE21 and KYSE450 was conducted in triplicate. RNA was extracted from untreated cells (RNeasy kit – Qiagen, Crawley, West Sussex, UK). RNA sample quality was tabulated by bioanalyser and biophotometric quality control criteria. *In vitro* cDNA synthesis, biotin labelling, transcription, fragmentation and hybridisation to the Affymetrix GeneChip Human Genome U133 Plus 2.0 array was carried out by Almac Diagnostics (Craigavon Co., Armagh, UK) (www.almac.com).

### RNA extraction and real-time PCR

RNA was extracted, treated with DNase (Ambion, Dublin, Ireland) and cDNA synthesised (Qiagen). Each real-time PCR reaction contained 0.5 *μ*M of each primer, MgCl_2_ (Noxa 4 mM, *β*-actin 3 mM), 1 × LightCycler FastStart DNA Master SYBR Green and template cDNA. Primers: NoxaF 5′-CTCCAGCAGAGCTGGAAGTC-3′, NoxaR 5′-GATGAAACGTGCACCTCCTGAG-3′, *β*-actinF 5′-GACCCAGATCATGTTTGAGA-3′, *β*-actinR 5′-CTTCATGAGGTAGTCAGTCA-3′. PCR conditions: 95 °C for 10 min; 50 × for Noxa (*β*-actin 30 × ) of 95 °C for 10 s, 62 °C for 5 s for Noxa (*β*-actin 57 °C), 72 °C for 7 s for Noxa (*β*-actin 8s) (Lightcycler System, Roche, Burgess Hill, West Sussex, UK). Relative *Noxa* transcript levels in the samples were quantified using the ‘Delta-Delta’ formula (Pfaffl, 2001).

### Immunofluorescence analysis

Cells were fixed in 4% paraformaldehyde and incubated with anti-LC3 (Abgent, Oxfordshire, UK) or anti-caspase 3 (Cell Signaling, Danvers, MA, USA) and AlexaFluor-488 secondary antibody with standard washing procedures (Invitrogen, Paisley, UK). Analysis was carried out using an Olympus Fluorescence (Southend-on-Sea, Essex, UK) microscope.

### Colony formation assay/statistical analysis

Colony formation assay determines whether cells can recover from treatment. Following treatment, viable cells were re-seeded in fresh media (without drug) in a six-well plate (in triplicate) and allowed to grow for 12–14 days. Colonies were fixed in 96% ethanol and stained with ProDiff solution C (Braidwood Laboratories) and subsequently counted (presented as mean±s.e.m.).

## Results

### Cell death induction with 5-fluorouracil

**Figure 1:**
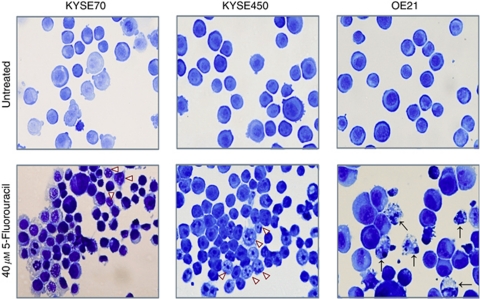
Effect of 5-FU on cell death morphology in oesophageal cancer cell lines. Following 5-FU treatment (40 *μ*M – 48 h), KYSE70 and KYSE450 cells display features of non-apoptotic cell death/type II cell death (arrowheads). OE21 cells display features of apoptosis (arrows) ( × 40 magnification).

We investigated the cellular response of oesophageal cancer cell lines to 5-FU (40 *μ*M) for 48 h and evaluated morphological features of apoptosis or non-apoptotic (type II) cell death (as described in the Materials and Methods section). The response of two of these cell lines (OE21 and KYSE450) to chemotherapeutics has previously been reported together with apoptosis and autophagy markers (O’Donovan *et al*, 2011). Following 5-FU treatment, KYSE70 (Figure 1, lower left panel) and KYSE450 (Figure 1, lower middle panel) cell lines exhibit predominantly type II cell death morphology (arrowheads), whereas OE21 cells (Figure 1, lower right panel) induce apoptosis (arrows). These death responses are not altered by extended incubations or drug concentration (O’Donovan *et al*, 2011).

### Bcl-2 family expression in cell lines

**Figure 2:**
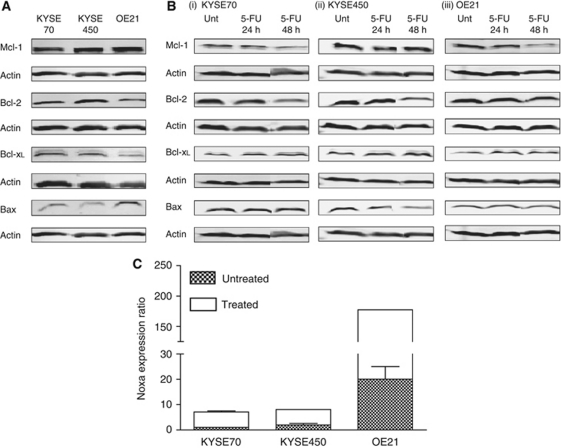
Bcl-2 family expression in oesophageal cancer cell lines. (**A**) Western blot analysis of basal levels of Mcl-1, Bcl-2, Bcl-x_L_ and Bax in KYSE70, KYSE450 and OE21 cells. (**B**) Mcl-1, Bcl-2, Bcl-x_L_ and Bax expression following treatment with 5-FU (40 *μ*M – 24 and 48 h) in (i) KYSE70, (ii) KYSE450 and (iii) OE21 cells. *β*-Actin was a loading control. (**C**) Basal expression levels of NOXA (hatched boxes) and levels following 5-FU treatment (40 *μ*M – 48 h, clear boxes) were determined by real-time PCR. *β*-Actin was used for normalisation. Differential expression was determined using the ΔΔCp formula, KYSE450 and OE21 cell lines were compared with KYSE70 cells, which is given an arbitrary value of 1. Error bars represent s.e.m.

The molecular determinants of apoptotic or autophagic responses to drug treatment in these cells are unknown. It is possible that high expression of negative regulators of apoptosis may impede apoptosis induction and autophagy is then induced as a default response to cellular damage. The concept of re-opening apoptotic signalling with a BH3 mimetic could therefore be explored, providing these Bcl-2 family members and a key positive effector of apoptosis (Bax or Bak) is expressed. We therefore evaluated basal expression levels of key Bcl-2 family members. The anti-apoptotic proteins Mcl-1, Bcl-2 and Bcl-x_L_ were expressed in all cell lines as was the pro-apoptotic protein Bax. Bcl-2 and Bcl-x_L_ expression was slightly lower in the OE21 (apoptosis-sensitive) cell line and Bax was slightly higher, suggesting that this imbalance may be important for apoptosis susceptibility (Figure 2A).

Expression was also evaluated following treatment with 5-FU (40 *μ*M) for 24 and 48 h (Figure 2B). There was no further loss of anti-apoptotic proteins in the OE21 cell line until 48 h when apoptosis would be initiated resulting in the degradation of many proteins. There was also no significant change in Bcl-2 family expression in the KYSE70 and KYSE450 cells.

### Expression and induction of *NOXA*

We undertook Affymetrix array analysis (GeneChip Human Genome U133 Plus 2.0 arrays) to compare gene expression patterns in two apoptosis competent (OE21 and OE33) and two apoptosis incompetent oesophageal cancer cell lines (KYSE450 and OE19) (cell lines previously described in (O’Donovan *et al*, 2011)). This analysis included two cell lines from this study (OE21 and KYSE450). Multi-domain Bcl-2 family members were not identified as being differentially expressed in the two groups. The only BH3-only protein flagged was NOXA. Noxa is a stress responsive BH3-only protein that can interact with Mcl-1 and with lower affinity to Bfl1/A1 to induce apoptosis (Ploner *et al*, 2008). We therefore analysed *NOXA* transcript levels in KYSE70, KYSE450 and OE21 cell lines as absence of *NOXA* expression could be a major factor in their failure to undergo apoptosis. Real-time PCR analysis indicated that KYSE70 and KYSE450 (which undergo autophagy) have lower basal *NOXA* expression. As *NOXA* is a damage inducible gene, we also evaluated expression following treatment with 5-FU. Although *NOXA* expression was inducible in all cell lines in response to 5-FU (∼4-fold) at 48 h, its expression in KYSE70 and KYSE450 was still well below the basal expression levels in OE21 cells (Figure 2C) suggesting there may be a deficiency in BH3-only signalling. We therefore evaluated the possibility of enhancing apoptosis with a mimetic that can inhibit the activities of Bcl-2 family members. HA14-1 is a small molecule inhibitor of Bcl-2 and has been shown to disrupt Bax and Bcl-2 interactions (Wang *et al*, 2000). HA14-1 has previously been shown to enhance apoptosis induction in various tumour cell lines (Kang and Reynolds, 2009).

### Effect of HA14-1 (BH3 mimetic) on cytotoxicity and recovery of drug-treated populations

**Figure 3:**
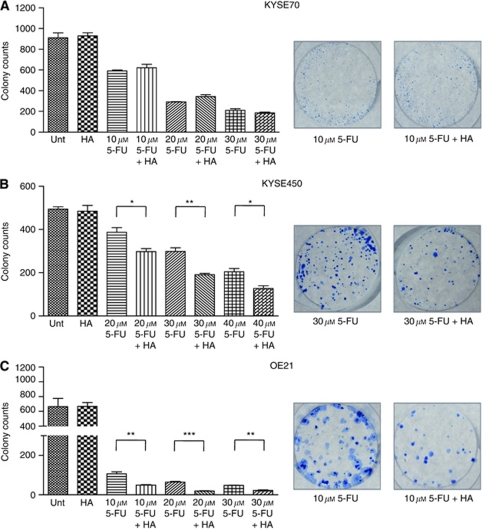
Effects of 5-FU or HA14-1 alone and in combination on clonogenic recovery of oesophageal cancer cells. (**A**) KYSE70 cells were treated with 5-FU (10–30 *μ*M) alone or in combination with HA14-1 (20 *μ*M) for 24 h. (**B**) KYSE450 cells were treated with 5-FU (20–40 *μ*M) alone or in combination with HA14-1 (20 *μ*M) for 48 h. (**C**) OE21 cells were treated with 5-FU (10–30 *μ*M) alone or in combination with HA14-1 (20 *μ*M) for 24 h. Drug-treated and control cells were counted and 2000 (KYSE70 and OE21 cells) or 1000 (KYSE450 cells) were re-seeded in triplicate wells without drug and allowed to grow for 12–14 days. Colonies were fixed and counted (presented as mean±s.e.m.). Representative wells are shown on the right-hand panels. Asterisks indicate statistical significance determined by *t*-test (^*^P<0.05, ^**^P<0.01, ^***^P<0.0001).

We have previously shown that cells that induce apoptosis (OE21) fail to recover from 5-FU treatment, but cells that induce autophagy (KYSE450) recover when the drug is withdrawn (O’Donovan *et al*, 2011). In this study, we evaluated the effects of HA14-1 on both cell viability at 24 and 48 h and the capacity of cells to recover in assays of clonogenic growth. Treatment times and drug concentrations were lowered in the more sensitive cell lines to achieve moderate recovery from 5-FU treatment alone and to enable comparison with combination treatment. All cell lines were treated with HA14-1 (20 *μ*M) in the presence and absence of 5-FU. As a single agent, HA14-1 (20 *μ*M) has minimal effects on clonogenic recovery in all cell lines (Figure 3).

KYSE70 cells were treated for 24 h at a range of concentrations of 5-FU (10–30 *μ*M). The combination of 5-FU (10–30 *μ*M) and HA14-1 (20 *μ*M) did not further sensitise these cells, nor did it alter their ability to recover compared with 5-FU alone (Figure 3A). KYSE450 cells are more drug resistant and show considerable recovery following 48-h treatment with 40 *μ*M 5-FU. The combination of 5-FU (20–40 *μ*M) and HA14-1 (20 *μ*M) significantly impeded the recovery of KYSE450 cells compared with 5-FU treatment alone (Figure 3B). OE21 is the most drug-sensitive cell line; however, when the duration of drug treatment is reduced to 24 h, limited recovery can be observed following treatment at 10, 20 and 30 *μ*M 5-FU. When 5-FU (10–30 *μ*M) and HA14-1 (20 *μ*M) are combined, OE21 cells are further sensitised and recovery is significantly reduced (Figure 3C).

It is important to note that following treatment with either 5-FU or HA14-1 alone or in combination, the numbers of viable cells (for both KYSE450 and OE21 cells) were not significantly reduced by the combination treatment at the 24- and 48-h time points (Supplementary figure); yet, their ability to recover and form colonies is compromised.

These data suggest that there are clear benefits to combining the chemotherapeutic 5-FU with HA14-1 in both the KYSE450 and OE21 cell lines; however this combination regime is of no benefit in KYSE70 cells.

### Effects of HA14-1 (BH3 mimetic) on apoptosis and type II cell death morphologies

**Figure 4:**
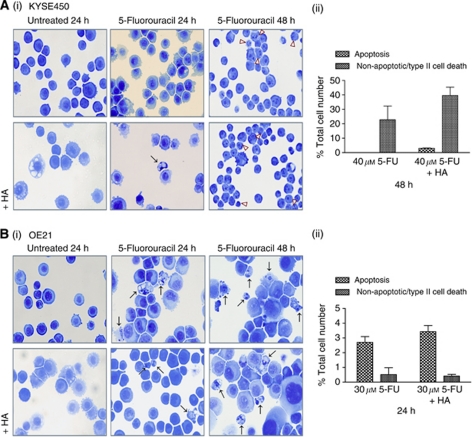
Effect of drug treatments on cell death morphology. (**A**(i)) Morphological features of KYSE450 cells treated with 5-FU (40 *μ*M) alone (upper panels) or in combination with HA14-1 (20 *μ*M) (lower panels) for 24 or 48 h. (**B**(i)) Morphological features of OE21 cells treated with 5-FU (30 *μ*M) alone (upper panels) or in combination with HA14-1 (20 *μ*M) (lower panels) for 24 or 48 h. Apoptosis and non-apoptotic/type II cell death are shown by arrows and arrowheads respectively ( × 40 magnification). The extent of type II (non-apoptotic) and apoptotic cell death in KYSE450 cells treated with 5-FU (40 *μ*M) alone and in combination with HA14-1 (20 *μ*M) for 48 h (**A**(ii)) and OE21 cells treated with 30 *μ*M 5-FU alone and in combination with HA14-1 (20 *μ*M) for 24 h (**B**(ii)) was determined by counting six fields of view per slide, with ∼350 cells per field ( × 10 magnification).

It is currently unclear whether HA14-1 re-activates apoptosis in previously apoptosis incompetent cells or induces an alternative death mechanism. We therefore looked for evidence of apoptosis, autophagy and type II cell death in the cell lines that respond to combination treatment. Cells were treated with HA14-1 (20 *μ*M) in the presence and absence of 5-FU for 24 and 48 h (Figure 4).

In KYSE450 cells, HA14-1 (20 *μ*M) alone resulted in minor accumulation of cytoplasmic vesicles (Figure 4A(i), lower left panel). At 24 h, 5-FU (40 *μ*M) alone induces mild accumulation of cytoplasmic vesicles (Figure 4A(i), upper middle panel) and the combination treatment with HA14-1 accentuates this morphology, with a rare appearance of an apoptotic cell (Figure 4A(i), lower middle panel). At 48 h, the predominant morphology in response to 5-FU (40 *μ*M) alone is non-apoptotic/type II cell death (Figure 4A(i), upper right panel), which is enhanced in combination with HA14-1 (20 *μ*M) (Figure 4A(i), lower right panel). These morphological features were quantified by counting cells treated for 48 h (40 *μ*M 5-FU±20 *μ*M HA14-1) (Figure 4A(ii)). These data demonstrate that the addition of HA14-1 to 5-FU treatment rarely induced apoptosis and the principal morphology in affected cells was type II cell death.

The more drug-sensitive OE21 cells respond to 5-FU treatment (30 *μ*M) by inducing apoptosis (Figure 4B(i), upper middle and right panel). HA14-1 (20 *μ*M) alone caused mild induction of cytoplasmic vesicles in a small number of cells (Figure 4B(i), lower left panel) but the combination treatment of both 5-FU and HA14-1 at 24 and 48 h, resulted in persistence of apoptosis (Figure 4B(i), lower middle and right panel and 4B(ii)). These morphological features were quantified by counting cells treated for 24 h (30 *μ*M 5-FU±20 *μ*M HA14-1) (Figure 4B(ii)). Levels of apoptosis were low at 24 h (<5%) and are not significantly different between 5-FU and the combination of 5-FU and HA14-1. However, the recovery of treated OE21 cells (previous section) was clearly affected, suggesting an augmented apoptotic death in the presence of HA14-1. Morphology at 48 h again shows predominance of apoptosis with similar levels in both 5-FU and the HA14-1 combination (∼30%). OE21 cells treated for 48 h with 5-FU (30 *μ*M) alone will not recover in clonogenic assays (data not shown). It is noteworthy that if exposure to 5-FU can be maintained for a sufficient length of time, there will be no benefit to adding HA14-1, as apoptosis will be induced in these cells by the chemotherapeutic alone.

### Markers of autophagy and apoptosis

**Figure 5:**
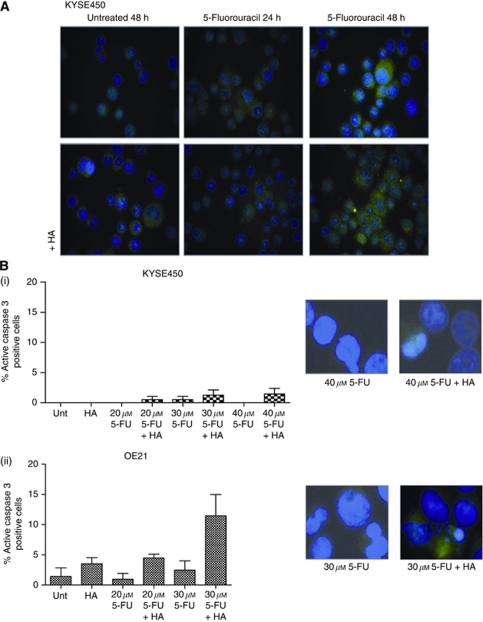
Effect of drug treatments on markers of autophagy and apoptosis. (**A**) LC3 immunofluorescence staining of KYSE450 cells treated with 5-FU (40 *μ*M) alone or in combination with HA14-1 (20 *μ*M) for 24 and 48 h. Untreated KYSE450 cells displayed a diffuse LC3 distribution (upper left panel), in contrast, following treatment, staining was bright and punctuate (all other panels). (**B**) The extent of active caspase 3 was analysed by fluorescence microscopy in (i) KYSE450 and (ii) OE21 cells treated with a range of concentrations of 5-FU (KYSE450 cells 20–40 *μ*M 5-FU; OE21 cells 20–30 *μ*M 5-FU) and HA14-1 (20 *μ*M) for 48 and 24 h, respectively. Cells exhibiting active caspase 3 were quantified by counting five fields of view per slide, with ∼30 cells per field ( × 40 magnification). Representative images of caspase 3 are shown on the right-hand panels.

To confirm the presence of early autophagy in KYSE450 cells, we examined LC3 distribution in drug-treated cells (Figure 5A). Both 5-FU (40 *μ*M) and HA14-1 (20 *μ*M) alone resulted in a punctuate distribution of LC3 indicating the formation of early autophagosomes. The combination of 5-FU and HA14-1 resulted in a modest enhancement of the number of cells with LC3 staining (Figure 5A, lower middle and right panel). In OE21 cells, HA14-1 (20 *μ*M) alone induced early features of autophagy, an effect that was confirmed by redistribution of LC3. However, LC3 staining was not enhanced in combination-treated cells (data not shown).

Levels of active caspase 3 were quantified in KYSE450 and OE21 cell lines (Figure 5B). There were no significant activation of caspase 3 in the KYSE450-treated cells (Figure 5B(i)). A modest increase in active caspase 3 was detected in the OE21 cells treated with the combination of 5-FU (20–30 *μ*M) and HA14-1 (20 *μ*M) (Figure 5B(ii)), which reflects apoptosis levels observed at 24 h (Figure 4B(ii)).

These data indicate that when cancer cells are apoptosis competent, HA14-1 can further enhance the early induction of this program. However, if 5-FU is present for sufficient time, apoptosis will be induced and may be adequate to prevent recovery of the cells. In apoptosis incompetent cells, there is either no response (KYSE70) or there is an enhancement of autophagy and type II cell death (KYSE450). Our current analysis of the expression of Bcl-2 family members could not predict which cell lines would respond and clearly an enhanced cell death response can be independent of apoptosis.

## Discussion

The design of BH3 mimetics was based on the rationale that by acting as inhibitors of the anti-apoptotic Bcl-2 family members, these compounds would activate apoptosis. However, it is currently unclear whether this is their mode of action in all cancer cells or whether they could also induce alternative forms of cell death. In addition, recent reports of interaction with autophagy regulators underscore the need for a re-evaluation of their activity – as autophagy can be both protective and detrimental to cell viability.

We have previously shown how cell death mechanisms can influence the chemotherapeutic response and recovery of oesophageal cancer cells (O’Donovan *et al*, 2011). In this study, we investigated whether the addition of a BH3 mimetic (HA14-1) could influence cellular response to 5-FU. In apoptosis competent OE21 cells, the combination of HA14-1 and 5-FU promoted early apoptosis and reduced clonogenic survival compared with 5-FU alone. Of the two apoptosis incompetent/type II cell death inducing cells lines, only KYSE450 was sensitised by the combination of HA14-1 and 5-FU, resulting in reduced recovery. The cell death observed in KYSE450 cells was type II and this was preceded by elevated autophagy. These data suggest that HA14-1 can enhance the toxicity of 5-FU in the absence of apoptosis by advancing autophagy and type II cell death. However, as is the case with KYSE70 cells, not all apoptosis incompetent/type II cells will be susceptible to HA14-1.

Other studies have reported that HA14-1 induces apoptosis in cancer cell lines. It has been shown to enhance the cytotoxicity of several compounds (Kang and Reynolds, 2009). The precise mechanism of action of HA14-1 is unclear. HA14-1 is an inhibitor of Bcl-2 and is thought to disrupt Bax and Bcl-2 interactions (Wang *et al*, 2000; Manero *et al*, 2006). Expression of Mcl-1 may impact on the effectiveness of HA14-1 but this is poorly understood and may be cell line dependent (Simonin *et al*, 2009). To the best of our knowledge, no study has examined the consequences of combination treatment of HA14-1 and 5-FU.

Several BH3 mimetics are currently in either pre-clinical development or have advanced to clinical trials (Zhang *et al*, 2007; Ghiotto *et al*, 2010). These include gossypol and its analogues (targets Bcl-2, Bcl-x_L_ and Mcl-1), GX15-070 (Obatoclax – Gemin X Biotechnologies (Montreal, QC, Canada), which binds to all anti-apoptotic Bcl-2 family members) and ABT-737 (binds to Bcl-2, Bcl-x_L_ and Bcl-w but not to Mcl-1). ABT-737 is one of the most advanced Bcl-2 inhibitors in clinical development (oral version is ABT-263 – Abbott Laboratories, Chicago, IL, USA). Cell lines and tumours expressing high Mcl-1 levels are resistant to ABT-737 (van Delft *et al*, 2006; Lessene *et al*, 2008). In lymphoblastic and small cell lung cancers, ABT-737 was found to enhance apoptosis (Leber *et al*, 2010). The majority of studies indicate that ABT-737 does not directly induce cell death and has limited value as a mono-therapy. It can, however, enhance susceptibility to death and has been shown to be synergistic with chemotherapeutics and radiation (Oltersdorf *et al*, 2005).

The precise mechanism of action of mimetics is unclear as some have weak affinities for their putative targets (Zhai *et al*, 2006). ABT-737 has also been shown to induce autophagy. It disrupts the interaction between Bcl-2/Bcl-x_L_ and Beclin 1, thereby releasing Beclin 1 to initiate autophagy (Maiuri *et al*, 2007). Both HA14-1 and ABT-737 have been reported to stimulate multiple pro-autophagic signal transduction pathways and to activate the nutrient sensors Sirtuin 1 and AMP-dependent kinase, inhibit mammalian target of rapamycin, deplete cytoplasmic p53 and trigger the IB kinase (Malik *et al*, 2011). A new analogue of HA14-1 (sHA 14-1) has also been reported to induce ER stress and calcium release, which may contribute to its mechanism of action (Hermanson *et al*, 2009). In keeping with these and our findings, recent reports have shown that treatment with HA14-1 alone can induce autophagy in leukaemic, osteosarcoma, ovarian and cervical cancer cell lines (Kessel and Reiners, 2007; Simonin *et al*, 2009; Malik *et al*, 2011).

HA14-1, gossypol and GX15-070 have been reported to promote caspase-independent cell death and do not require Bax/Bak for their cytotoxicity (van Delft *et al*, 2006; Ghiotto *et al*, 2010). In the MCF-7 cell line model, gossypol (a natural BH3 mimetic) induced both Beclin 1 dependent and Beclin 1-independent cytoprotective autophagy (Gao *et al*, 2010). In contrast, in malignant glioma cells gossypol potentiated the cell death induced by temozolomide and autophagy contributed to this type of cell death (Voss *et al*, 2010). Also, analysis of androgen-independent prostate cancer cells showed that gossypol interrupted Beclin 1 and Bcl-2/Bcl-x_L_ interactions and that gossypol-induced autophagy was dependent on Beclin 1 and Atg5 (Lian *et al*, 2011).

In acute lymphoblastic and acute myeloid leukaemia cells, combination treatment with GX15-070 activated both apoptosis and autophagy (Heidari *et al*, 2010; Wei *et al*, 2010). In non-small cell lung cancer cell line models, GX15-070-induced Atg7-dependent autophagy independent of Beclin 1 and Bax/Bak (McCoy *et al*, 2010). GX15-070 has been used in combination with chemotherapeutics for *in vitro* studies of oesophageal cancer. It has been reported that GX15-070-induced autophagy and inhibited the growth of oesophageal cancer cells. It was also found to synergise with either carboplatin or 5-FU through enhanced apoptosis. In their model, inhibiting autophagy increased the levels of apoptosis suggesting that autophagy induced by GX15-070 treatment may have a cyto-protective effect (Pan *et al*, 2010). This is in contrast to this study in which we found that combining HA14-1 with 5-FU increased the levels of autophagy and subsequent type II cell death suggesting that the observed reduction in clonogenic survival in KYSE450 cells is due to the enhancement of this form of cell death.

Collectively these studies indicate that activation of apoptotic signalling cascade may not be the sole activity of BH3 mimetics. In our study, combining HA14-1 with 5-FU in KYSE450 cells resulted in enhanced autophagy and type II cell death. However, not all chemo-resistant cells (KYSE70) benefited from this type of treatment and a deficiency of the DNA damage responsive BH3-only protein-Noxa was not predictive of response. Clearly, unless we understand the molecular determinants of response, these compounds will be used clinically without adequate predictive markers. The consequences of autophagy induction also seem to vary with some studies, like ours, finding enhanced type II cell death, whereas others report a protective effect.

In this study, HA14-1 was found to be of benefit in 2 out of 3 oesophageal cancer cell lines when used in conjugation with 5-FU. Induction of apoptosis was not required for chemo-sensitisation. It could be argued that apoptosis competent tumours will be more treatable with chemotherapeutics alone and that the real benefit of these mimetics may be in enhancing type II cell death in apoptosis incompetent cells. The combination of HA14-1 and 5-FU could be a potential treatment for oesophageal cancer if the problems pertaining to HA14-1's poor solubility and stability could be overcome. Newer analogues are already showing promise in this regard (Tian *et al*, 2008). In addition, molecular markers truly predictive of response need to be established. Alternatively, combining 5-FU with a more potent inducer of type II cell death may be an alternative treatment strategy for oesophageal cancer.

## References

[bib1] Adams JM, Cory S (2007) The Bcl-2 apoptotic switch in cancer development and therapy. Oncogene 26: 1324–1337 17322918 10.1038/sj.onc.1210220 PMC2930981

[bib2] Berry DL, Baehrecke EH (2007) Growth arrest and autophagy are required for salivary gland cell degradation in Drosophila. Cell 131: 1137–1148 18083103 10.1016/j.cell.2007.10.048 PMC2180345

[bib3] Clarke PG (1990) Developmental cell death: morphological diversity and multiple mechanisms. Anat Embryol (Berl) 181: 195–213 2186664 10.1007/BF00174615

[bib4] Gao P, Bauvy C, Souquere S, Tonelli G, Liu L, Zhu Y, Qiao Z, Bakula D, Proikas-Cezanne T, Pierron G, Codogno P, Chen Q, Mehrpour M (2010) The Bcl-2 homology domain 3 mimetic gossypol induces both Beclin 1-dependent and Beclin 1-independent cytoprotective autophagy in cancer cells. J Biol Chem 285: 25570–25581 20529838 10.1074/jbc.M110.118125 PMC2919121

[bib5] Ghiotto F, Fais F, Bruno S (2010) BH3-only proteins: the death-puppeteer's wires. Cytometry A 77: 11–21 19899133 10.1002/cyto.a.20819

[bib6] Hamacher-Brady A, Brady NR, Logue SE, Sayen MR, Jinno M, Kirshenbaum LA, Gottlieb RA, Gustafsson AB (2007) Response to myocardial ischemia/reperfusion injury involves Bnip3 and autophagy. Cell Death Differ 14: 146–157 16645637 10.1038/sj.cdd.4401936

[bib7] Heidari N, Hicks MA, Harada H (2010) GX15-070 (obatoclax) overcomes glucocorticoid resistance in acute lymphoblastic leukemia through induction of apoptosis and autophagy. Cell Death Dis 1: e76 21364679 10.1038/cddis.2010.53 PMC3032343

[bib8] Hermanson D, Addo SN, Bajer AA, Marchant JS, Das SG, Srinivasan B, Al-Mousa F, Michelangeli F, Thomas DD, Lebien TW, Xing C (2009) Dual mechanisms of sHA 14-1 in inducing cell death through endoplasmic reticulum and mitochondria. Mol Pharmacol 76: 667–678 19561125 10.1124/mol.109.055830 PMC2730395

[bib9] Kang MH, Reynolds CP (2009) Bcl-2 inhibitors: targeting mitochondrial apoptotic pathways in cancer therapy. Clin Cancer Res 15: 1126–1132 19228717 10.1158/1078-0432.CCR-08-0144 PMC3182268

[bib10] Kessel D, Reiners Jr JJ (2007) Initiation of apoptosis and autophagy by the Bcl-2 antagonist HA14-1. Cancer Lett 249: 294–299 17055152 10.1016/j.canlet.2006.09.009 PMC1924967

[bib11] Lamparska-Przybysz M, Gajkowska B, Motyl T (2005) Cathepsins and BID are involved in the molecular switch between apoptosis and autophagy in breast cancer MCF-7 cells exposed to camptothecin. J Physiol Pharmacol 56(Suppl 3): 159–179 16077201

[bib12] Leber B, Geng F, Kale J, Andrews DW (2010) Drugs targeting Bcl-2 family members as an emerging strategy in cancer. Expert Rev Mol Med 12: e28 20822554 10.1017/S1462399410001572

[bib13] Lessene G, Czabotar PE, Colman PM (2008) BCL-2 family antagonists for cancer therapy. Nat Rev Drug Discov 7: 989–1000 19043450 10.1038/nrd2658

[bib14] Lian J, Wu X, He F, Karnak D, Tang W, Meng Y, Xiang D, Ji M, Lawrence TS, Xu L (2011) A natural BH3 mimetic induces autophagy in apoptosis-resistant prostate cancer via modulating Bcl-2-Beclin1 interaction at endoplasmic reticulum. Cell Death Differ 18: 60–71 20577262 10.1038/cdd.2010.74 PMC2950895

[bib15] Maiuri MC, Le Toumelin G, Criollo A, Rain JC, Gautier F, Juin P, Tasdemir E, Pierron G, Troulinaki K, Tavernarakis N, Hickman JA, Geneste O, Kroemer G (2007) Functional and physical interaction between Bcl-X(L) and a BH3-like domain in Beclin-1. EMBO J 26: 2527–2539 17446862 10.1038/sj.emboj.7601689 PMC1868901

[bib16] Malik SA, Orhon I, Morselli E, Criollo A, Shen S, Marino G, Benyounes A, Benit P, Rustin P, Maiuri MC, Kroemer G (2011) BH3 mimetics activate multiple pro-autophagic pathways. Oncogene 30: 3918–3929 21460857 10.1038/onc.2011.104

[bib17] Manero F, Gautier F, Gallenne T, Cauquil N, Gree D, Cartron PF, Geneste O, Gree R, Vallette FM, Juin P (2006) The small organic compound HA14-1 prevents Bcl-2 interaction with Bax to sensitize malignant glioma cells to induction of cell death. Cancer Res 66: 2757–2764 16510597 10.1158/0008-5472.CAN-05-2097

[bib18] McCoy F, Hurwitz J, McTavish N, Paul I, Barnes C, O’Hagan B, Odrzywol K, Murray J, Longley D, McKerr G, Fennell DA (2010) Obatoclax induces Atg7-dependent autophagy independent of beclin-1 and BAX/BAK. Cell Death Dis 1: e108 21368880 10.1038/cddis.2010.86 PMC3032298

[bib19] O’Donovan TR, O’Sullivan GC, McKenna SL (2011) Induction of autophagy by drug-resistant esophageal cancer cells promotes their survival and recovery following treatment with chemotherapeutics. Autophagy 7: 509–524 21325880 10.4161/auto.7.6.15066 PMC3127212

[bib20] Oltersdorf T, Elmore SW, Shoemaker AR, Armstrong RC, Augeri DJ, Belli BA, Bruncko M, Deckwerth TL, Dinges J, Hajduk PJ, Joseph MK, Kitada S, Korsmeyer SJ, Kunzer AR, Letai A, Li C, Mitten MJ, Nettesheim DG, Ng S, Nimmer PM, O’Connor JM, Oleksijew A, Petros AM, Reed JC, Shen W, Tahir SK, Thompson CB, Tomaselli KJ, Wang B, Wendt MD, Zhang H, Fesik SW, Rosenberg SH (2005) An inhibitor of Bcl-2 family proteins induces regression of solid tumours. Nature 435: 677–681 15902208 10.1038/nature03579

[bib21] Pan J, Cheng C, Verstovsek S, Chen Q, Jin Y, Cao Q (2010) The BH3-mimetic GX15-070 induces autophagy, potentiates the cytotoxicity of carboplatin and 5-fluorouracil in esophageal carcinoma cells. Cancer Lett 293: 167–174 20153924 10.1016/j.canlet.2010.01.006

[bib22] Pfaffl MW (2001) A new mathematical model for relative quantification in real-time RT-PCR. Nucleic Acids Res 29: e45 11328886 10.1093/nar/29.9.e45 PMC55695

[bib23] Ploner C, Kofler R, Villunger A (2008) Noxa: at the tip of the balance between life and death. Oncogene 27(Suppl 1): S84–S92 19641509 10.1038/onc.2009.46 PMC3272398

[bib24] Simonin K, Brotin E, Dufort S, Dutoit S, Goux D, N′Diaye M, Denoyelle C, Gauduchon P, Poulain L (2009) Mcl-1 is an important determinant of the apoptotic response to the BH3-mimetic molecule HA14-1 in cisplatin-resistant ovarian carcinoma cells. Mol Cancer Ther 8: 3162–3170 19887550 10.1158/1535-7163.MCT-09-0493

[bib25] Tian D, Das SG, Doshi JM, Peng J, Lin J, Xing C (2008) sHA 14-1, a stable and ROS-free antagonist against anti-apoptotic Bcl-2 proteins, bypasses drug resistances and synergizes cancer therapies in human leukemia cell. Cancer Lett 259: 198–208 18037229 10.1016/j.canlet.2007.10.012 PMC2693013

[bib26] van Delft MF, Wei AH, Mason KD, Vandenberg CJ, Chen L, Czabotar PE, Willis SN, Scott CL, Day CL, Cory S, Adams JM, Roberts AW, Huang DC (2006) The BH3 mimetic ABT-737 targets selective Bcl-2 proteins and efficiently induces apoptosis via Bak/Bax if Mcl-1 is neutralized. Cancer Cell 10: 389–399 17097561 10.1016/j.ccr.2006.08.027 PMC2953559

[bib27] Voss V, Senft C, Lang V, Ronellenfitsch MW, Steinbach JP, Seifert V, Kogel D (2010) The pan-Bcl-2 inhibitor (-)-gossypol triggers autophagic cell death in malignant glioma. Mol Cancer Res 8: 1002–1016 20587533 10.1158/1541-7786.MCR-09-0562

[bib28] Wan G, Zhaorigetu S, Liu Z, Kaini R, Jiang Z, Hu CA (2008) Apolipoprotein L1, a novel Bcl-2 homology domain 3-only lipid-binding protein, induces autophagic cell death. J Biol Chem 283: 21540–21549 18505729 10.1074/jbc.M800214200 PMC2490785

[bib29] Wang JL, Liu D, Zhang ZJ, Shan S, Han X, Srinivasula SM, Croce CM, Alnemri ES, Huang Z (2000) Structure-based discovery of an organic compound that binds Bcl-2 protein and induces apoptosis of tumor cells. Proc Natl Acad Sci USA 97: 7124–7129 10860979 10.1073/pnas.97.13.7124 PMC16510

[bib30] Wei Y, Kadia T, Tong W, Zhang M, Jia Y, Yang H, Hu Y, Tambaro FP, Viallet J, O’Brien S, Garcia-Manero G (2010) The combination of a histone deacetylase inhibitor with the Bcl-2 homology domain-3 mimetic GX15-070 has synergistic antileukemia activity by activating both apoptosis and autophagy. Clin Cancer Res 16: 3923–3932 20538760 10.1158/1078-0432.CCR-10-0032 PMC4113507

[bib31] Zhai D, Jin C, Satterthwait AC, Reed JC (2006) Comparison of chemical inhibitors of antiapoptotic Bcl-2-family proteins. Cell Death Differ 13: 1419–1421 16645636 10.1038/sj.cdd.4401937

[bib32] Zhang L, Ming L, Yu J (2007) BH3 mimetics to improve cancer therapy; mechanisms and examples. Drug Resist Update 10: 207–217 10.1016/j.drup.2007.08.002 PMC2265791 17921043

