# Supplementary figures and images for: The BH3 mimetic HA14-1 enhances 5-fluorouracil-induced autophagy and type II cell death in oesophageal cancer cells
Source: Br J Cancer. 2012 Jan 12;106(4):711–8. doi: 10.1038/bjc.2011.604 (PMC3322956; doi:10.1038/bjc.2011.604)

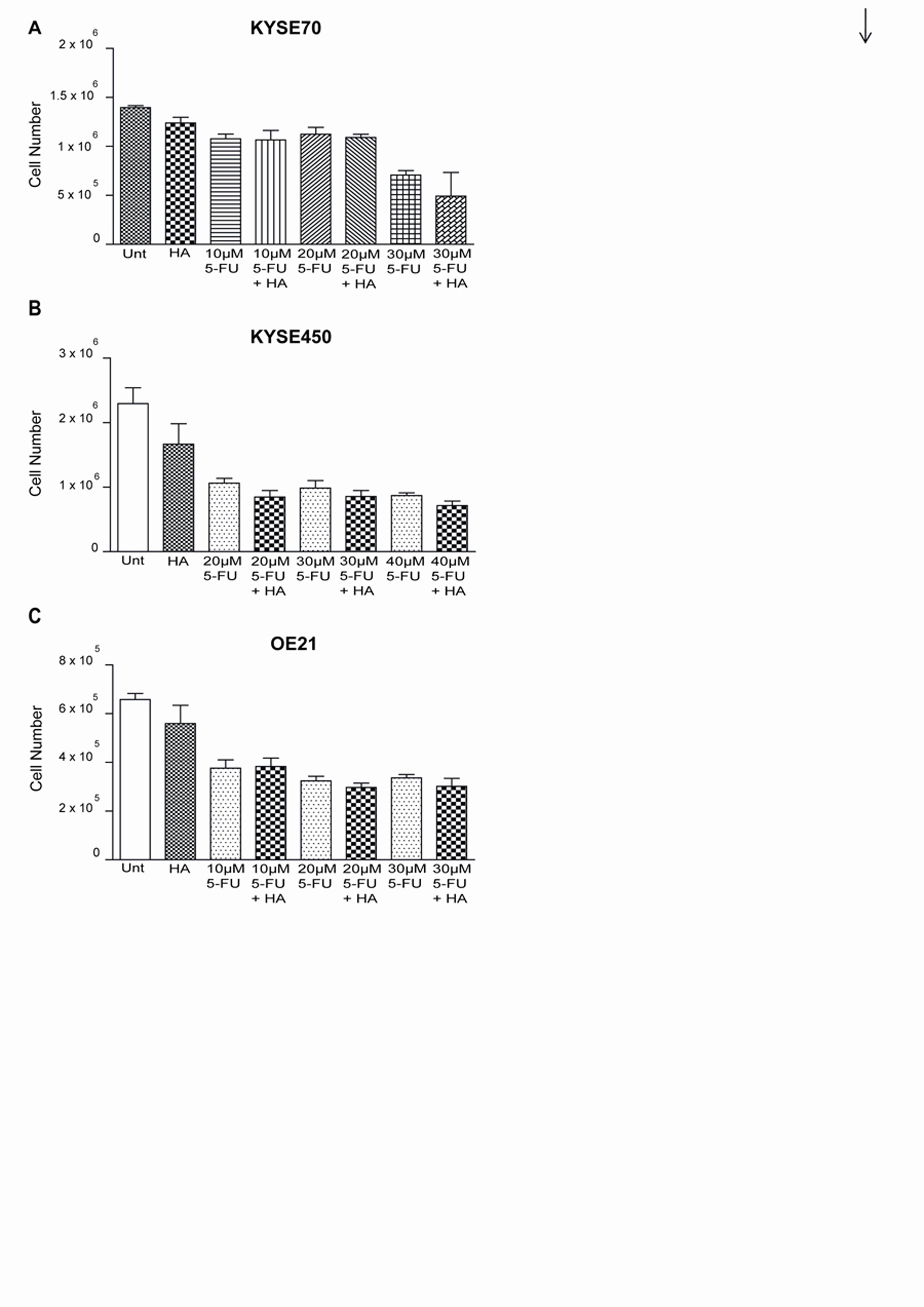

Supplement: Supplementary Figure [file bjc2011604x1.tif]
